# Short-course antimicrobial therapy for paediatric respiratory infections (SAFER): study protocol for a randomized controlled trial

**DOI:** 10.1186/s13063-018-2457-2

**Published:** 2018-02-01

**Authors:** Jeffrey Pernica, Stuart Harman, April Kam, Jacob Bailey, Redjana Carciumaru, Sarah Khan, Martha Fulford, Lehana Thabane, Robert Slinger, Cheryl Main, Marek Smieja, Mark Loeb

**Affiliations:** 1 Division of Infectious Disease, Department of Pediatrics, McMaster University, 1280 Main Street West, Hamilton, ON L8S 4K1 Canada; 2 Division of Emergency Medicine, Department of Pediatrics, University of Ottawa, 401 Smyth Road, Ottawa, ON K1H 8L1 Canada; 3 Division of Emergency Medicine, Department of Pediatrics, McMaster University, Hamilton, ON Canada; 4 Faculty of Health Sciences, McMaster University, Hamilton, ON Canada; 5 Department of Pediatrics, McMaster University, Hamilton, ON Canada; 6 Division of Infectious Disease, Department of Medicine, McMaster University, Hamilton, ON Canada; 7 Department of Health Research Methods, Evidence, and Impact, Hamilton, ON Canada; 8 Department of Pathology and Laboratory Medicine, University of Ottawa, Ottawa, ON Canada; 9 Department of Pathology and Molecular Medicine, McMaster University, Hamilton, ON Canada

**Keywords:** Community-acquired pneumonia, Antimicrobial stewardship, Amoxicillin, Microbiome, Respiratory virus, *Streptococcus pneumoniae*

## Abstract

**Background:**

Community-acquired pneumonia (CAP) is commonly diagnosed in children. The Infectious Disease Society of America guidelines recommend 10 days of high-dose amoxicillin for the treatment of non-severe CAP but 5-day “short course” therapy may be just as effective. Randomized trials in adults have already demonstrated non-inferiority of 5-day short-course treatment for adults hospitalized with severe CAP and for adults with mild CAP treated as outpatients. Minimizing exposure to antimicrobials is desirable to avoid harms including diarrhoea, rashes, severe allergic reactions, increased circulating antimicrobial resistance, and microbiome disruption.

**Methods:**

The objective of this multicentre, randomized, non-inferiority, controlled trial is to investigate whether 5 days of high-dose amoxicillin is associated with lower rates of clinical cure 14–21 days later as compared to 10 days of high-dose amoxicillin, the reference standard. Recruitment and enrolment will occur in the emergency departments of McMaster Children’s Hospital and the Children’s Hospital of Eastern Ontario. All children in the study will receive 5 days of amoxicillin after which point they will receive either 5 days of a different formulation of amoxicillin or a placebo. Assuming a clinical failure rate of 5% in the reference arm, a non-inferiority margin of 7.5%, one-sided alpha set at 0.025 and power of 0.80, 270 participants will be required. Participants from a previous feasibility study (n = 60) will be rolled over into the current study. We will be performing multiplex respiratory virus molecular testing, quantification of nasopharyngeal pneumococcal genomic loads, salivary inflammatory marker testing, and faecal microbiome profiling on participants.

**Discussion:**

This is a pragmatic study seeking to provide high-quality evidence for front-line physicians evaluating children presenting with mild CAP in North American emergency departments in the post-13-valent pneumococcal, conjugate vaccine era. High-quality evidence supporting the non-inferiority of short-course therapy for non-severe paediatric CAP should be generated prior to making changes to established guidelines.

**Trial registration:**

ClinicalTrials.gov, NCT02380352. Registered on 2 March 2015.

**Electronic supplementary material:**

The online version of this article (10.1186/s13063-018-2457-2) contains supplementary material, which is available to authorized users.

## Background

Respiratory infection is the leading cause of death in children worldwide [1, 2] and up to 5% of preschoolers in North America and Europe develop community-acquired pneumonia (CAP) every year [3, 4]. In August 2011, comprehensive guidelines for the diagnosis and treatment of paediatric CAP were published by the Infectious Disease Society of America (IDSA) [5]; definitive recommendations for the optimal duration of therapy could not be made due to a paucity of evidence. This guideline states that “Treatment courses of 10 days have been best studied [6], but shorter courses may be just as effective, particularly for more mild disease managed on an outpatient basis.” [5]. In contrast, in adults there is good evidence that 5 days of therapy is as effective as 7–10 days even in adults hospitalized because of CAP [7, 8], and so 5 days of therapy is generally recommended [9–11]. A recent survey of Canadian providers showed that 50% of all emergency department (ED)-based physicians using β-lactams treat mild paediatric pneumonia with 10 or more days of therapy [12].

Few trials have compared long-course (10-day) and short-course (<7-day) therapy for paediatric CAP. In 1994–95, Harris et al. randomized 456 children with CAP at 23 different US centres to either a 5-day course of azithromycin or to a 10-day regimen of either erythromycin or amoxicillin/clavulanate [13]. While the 5-day and 10-day arms were found to have similar success rates, macrolides are no longer the reference standard due to the increased prevalence of macrolide-resistant pneumococci today [14–16]. Moreover, because the half-life of azithromycin is 68 hours, a 5-day course of azithromycin is in effect much longer than a 5-day course of most β-lactams (half-life ~ 2 hours), so inferences about the potential success rate of short-course β-lactam therapy cannot be made on the basis of this trial. A recent randomized study in Israel compared 3-day, 5-day, and 10-day courses of amoxicillin therapy in preschool children aged 6–59 months with CAP [17]. They identified an increased failure rate in the 3-day group but no difference between the 5-day and 10-day groups. Unfortunately, the definition of the primary outcome, clinical cure, was relatively subjective and there were no clinical failures in either arm of the study; such an occurrence in a non-inferiority study removes all protection against bias afforded by blinding. Furthermore, the trial was stopped early for benefit, which has been demonstrated to be associated with exaggerated estimates of treatment effect [18]. World Health Organization (WHO) guidelines recommend 3 days of antibiotics for the treatment of non-severe pneumonia diagnosed in resource-limited settings [19], given that a systematic review of three trials (conducted in India, Pakistan, and Indonesia/Bangladesh) did not discern any increase in treatment failure or relapse rate in children treated with 3 as compared to 5 days of antibiotics [20]. However, it should be emphasized that the WHO diagnosis of pneumonia is fulfilled when a child has tachypnoea and either cough or difficulty breathing, with no exclusions for wheeze (or a separate diagnostic category for bronchiolitis), no requirement for fever, and no consideration for chest radiograph findings; consequently, these practice guidelines and evidence from the aforementioned trials are not generalizable to children diagnosed with CAP in upper-income countries.

In this age of increasing antibiotic resistance, the lack of evidence pertaining to optimal duration of treatment for paediatric CAP should be cause for concern. Optimizing antimicrobial prescribing, otherwise known as antimicrobial stewardship, has been noted to be the main strategy to deal with escalating antimicrobial resistance and has been called “a fiduciary responsibility for all healthcare institutions across the continuum of care” [21]. Many evidence-based guidelines published by Canadian and American authorities in the past ten years have sought to minimize the duration of systemic antimicrobials prescribed to both children and adults for the treatment of common infections [22–24]. Beyond the threat of antibiotic resistance, recent evidence suggests that there is association between the use of antibiotics and the development of obesity [25–32] and/or allergy [33, 34]. Antibiotic treatment has been observed to directly cause obesity in animal models, which appears in many cases to be mediated through effects on the intestinal microbiome [25–27]. Observational data suggest that this same relationship may be present in humans, with a greater magnitude of effect seen in younger age groups, especially with repeated antimicrobial courses [29, 30, 35].

Our principal research question is: in previously healthy children diagnosed with community-acquired pneumonia in the ED who are well enough to be treated as outpatients, does 5 days of oral high-dose amoxicillin lead to non-inferior rates of clinical cure at 14–21 days post-enrolment compared with the current standard, 10 days of oral high-dose amoxicillin? Our secondary research question is: are there any covariates – such as the detection of a respiratory virus/atypical pathogen, the pneumococcal genomic load in the nasopharynx, the C-reactive protein level, or a specific finding on chest radiograph – that modify the observed effectiveness of short-course antimicrobial treatment for mild paediatric CAP?

### Objectives

#### Primary

The primary outcome is to determine, in children diagnosed with mild CAP in the paediatric ED, whether 5 days of high-dose amoxicillin leads to non-inferior rates of clinical cure at day 14–21 compared to the reference standard of 10 days of high-dose amoxicillin.

#### Secondary

The secondary objectives relate to the secondary outcomes and include assessment of whether the 5-day course is: (1) non-inferior to the 10-day course with respect to either participant or caregiver absenteeism (from daycare/school or work, respectively); (2) associated with lower rates of mild drug adverse reactions, anaphylaxis, or other severe drug adverse reactions than the 10-day course; (3) associated with superior adherence to study medications than the 10-day course; (4) non-inferior to the 10-day course with respect to recurrence of respiratory illness within 30 days of cure; and (5) associated with lower rates of antibiotic-resistant organism (ARO) colonization and/or disruption of the gut microbiome than the 10-day course.

#### Tertiary

The tertiary objectives are to evaluate the following epidemiological features in children diagnosed with mild CAP in the current era of universal vaccination with the 13-valent pneumococcal vaccine (PCV13) and include:

1. To investigate various baseline characteristics in a cohort of children meeting study criteria for CAP, including the distribution of saliva C-reactive protein (CRP) values, the prevalence of *Streptococcus pneumoniae* high-level colonization (>10 000 genome copies/mL), in nasopharyngeal swab (NPS) specimens, how frequently *Mycoplasma pneumoniae* is detected in NPS samples, and what proportion of study participants with alveolar consolidation documented on chest radiograph have NPS specimens positive for at least one virus.
2. To investigate whether these baseline characteristics differ substantially when stratified by age group (6–59 months vs. 5–10 years of age).
3. To explore whether any of these baseline characteristics appear to be more common in children who do not achieve early clinical cure.

#### Subgroup

We will also explore whether there are differential treatment effects of the 5-day course vs. the 10-day course on the primary outcome of cure in the following subgroups: (1) older (age 5–10 years) vs. younger (age <5 years); (2) higher vs. lower salivary CRP; and (3) virus/*mycoplasma* detected in baseline NPS vs. no virus detected.

## Methods

### Study design and setting

SAFER is a multicentre, randomized, controlled, double-blind trial. The study will take place in the ED of McMaster Children’s Hospital (MCH, Hamilton, ON, Canada) and the Children’s Hospital of Eastern Ontario (CHEO, Ottawa, ON, Canada), both academic tertiary-care children’s hospitals.

### Study population

The trial will recruit previously well children aged 6 months to 10 years presenting with presumed CAP to the EDs of MCH and the CHEO located in Ottawa, ON, Canada.

### Inclusion criteria

Children aged 6 months to 10 years presenting with CAP will be eligible. Similar to other trials [16–19], CAP will be defined if all of the four following numeric criteria are met:

1. Fever (>37.5 °C axillary, >37.7 °C oral, or >38 °C rectal temperature) recorded in the ED or at home in the 48 h prior to presentation.
2. Any one of
  - Tachypnoea on examination (>60 breaths per minute (bpm) at age <1 year, > 50 bpm at age 1–2 years, > 40 bpm at age 2–4 years, and > 30 bpm at age >4 years);
  - Cough on examination or history of cough
  - Increased work of breathing on examination (characterized by the presence of scalene muscle use, or suprasternal recessions/in-drawing, or intercostal retractions, or subcostal recessions/in-drawing); or
  - Auscultatory findings (focal crackles, bronchial breathing, etc.) consistent with pneumonia.
3. Infiltrates on chest radiograph consistent with bacterial CAP as judged by the ED physician.
4. The attending ED physician diagnoses the child with primary CAP (children treated with systemic steroids in the ED will be presumed to have primary asthma exacerbation with possible infection and therefore will not meet inclusion criteria).

To be included, participants must be well enough to be treated as outpatients (adequate volume status, able to tolerate oral medication, oxygen saturation >90%, no evidence of impending respiratory failure); if a child is ill enough to be admitted to hospital, it might be unwise to attempt short-course therapy. Additionally, eligible participants must have no evidence of empyema or necrotizing pneumonia, as routine management of these conditions would require parenteral antibacterial agents (and admission to hospital).

### Exclusion criteria

Children will be excluded if they have any of the following conditions that would predispose to severe disease and/or pneumonia of unusual aetiology: cystic fibrosis, anatomic lung disease, bronchiectasis, congenital heart disease, history of repeated aspiration or velopharyngeal incompetence, malignancy, conditions requiring treatment with immune suppressants, primary immunodeficiency, advanced HIV infection, or renal dysfunction. Those with suspected infectious mononucleosis will be excluded because of the high risk of adverse events (i.e. rash) with amoxicillin treatment. Those having received > 24 hours of beta-lactam antibiotic therapy at presentation to the ED, at least a 5-day course of amoxicillin < 72 h prior to presenting to the ED, or an intravenous cephalosporin or azithromycin in the ED will not be eligible because it would be essentially impossible to give such children “short-course” treatment. Children will not be eligible to participate more than once. The Canadian product monograph for amoxicillin has a precaution against co-administration with warfarin or tetracyclines, so children receiving those drugs or anticoagulant therapy will be excluded. We will also exclude children with prolonged admission (>48 h) to hospital in the prior 2 months, pneumonia diagnosed in the prior month, or lung abscess in the prior 6 months, as we do not wish to enroll children with healthcare-acquired or complicated pneumonia. Finally, we will not recruit children with penicillin allergy.

### Study interventions

All study participants will begin the study receiving high-dose amoxicillin divided into three times daily and given orally for 5 days. Doses will vary over a range of 75–100 mg/kg/day within weight strata to simplify medication administration and reduce potential dosing errors. After the first 5 days of amoxicillin, half of the participants will take a second 5 days of amoxicillin dosed identically to the first 5 days (using a different product with a different flavour), and the other half of the participants will be given 5 days of placebo. This placebo will be Ora-Plus (NDC0574-0303-16) produced by Perrigo and distributed by Medisca, mixed with banana flavouring and sugar. No dose adjustments will be made.

### Study endpoints

#### Primary outcome

The primary outcome, early clinical cure, will be defined by meeting all of the following criteria:

1. Significant improvement in dyspnoea and increased work of breathing, and no recorded tachypnoea, at the day 14–21 follow-up visit
2. No more than one fever spike (as defined above) as a result of bacterial respiratory illness from day 4 up to and including the day 14–21 follow-up visit
3. Lack of a requirement for additional antibacterials or admission to hospital because of persistent/progressive lower respiratory illness during the 2 weeks after enrolment

This definition of clinical cure is similar to that used in other studies of 5-day CAP therapy in children [13] and adults [7]. Our definition was created using explicit criteria to ensure transparency and maximize the generalizability of the results. However, to optimize the appropriateness of the definition, the criteria are somewhat complex; this is to ensure that “failure” in the trial would be associated with a clinical scenario that would merit a change in overall management, even if that change was as little as a requirement for additional follow up by the treating clinician. The first criterion of the definition states “significant improvement” in respiratory symptoms; this will be assumed to be present if the participant’s caregiver opines that the child has no functional limitation resulting from any residual dyspnoea/increased work of breathing. The second criterion notes that more than a single spike of fever is required for “failure” to avoid erroneous conclusions resulting from an errant thermometer reading. Fever of unknown aetiology will be presumed to be associated with bacterial respiratory illness, but participants with fever due to other discernible causes, whether viral (new respiratory illness documented by a nasopharyngeal specimen positive for a virus not present at the time of initial enrolment, clinical croup, stomatitis/herpangina, hand-foot-mouth disease, gastroenteritis with positive stool results, conjunctivitis, meningoencephalitis, viral hip synovitis, perimyocarditis or myocarditis, hepatitis) or bacterial (cellulitis and other soft tissue infections, septic arthritis/osteomyelitis, meningitis, urinary tract infection with positive urinalysis, or cholecystitis) would be considered to have met clinical cure criteria. Clearly, admission to hospital – even if antimicrobial treatment does not need to be changed – is not conducive to short-course therapy and merits a decision of treatment “failure”.

For the measurement of the primary outcome, a physician or nurse, blinded to treatment allocation, will assess temperature, respiratory rate, and evident increased work of breathing, in person using standardized protocols; these physical examination findings are the most important when assessing response to therapy. Lack of fever at home will be verified through assessment of the symptom diaries. Medical visits for persistent respiratory illness will be assessed by directly asking the participant’s caregiver; though caregiver report is not an entirely reliable modality, we believe that the sensitivity and specificity of the question “Did your child see another health professional because of a concern about respiratory illness within the past ten days?” should be adequate.

#### Secondary outcomes

Secondary outcomes will include:

1. The number of days the participant is absent from school
2. The total number of caregiver days that their work is disrupted to care for the child
3. The number of days of mild drug adverse reactions
4. The incidence of severe drug adverse reactions (including anaphylaxis)
5. Participant adherence to the study medications, and
6. Recurrence of respiratory illness after the primary outcome visit in the month after enrolment that leads to an ED visit or another antimicrobial course, and
7. Development of new antibiotic-resistant organism colonization and the degree of perturbation of the intestinal microbiome at day 14–21 post-enrolment and 3–6 months post-enrolment
8. Determination of whether clinical cure rates are affected by baseline salivary C-reactive protein (CRP), baseline high-level *S. pneumoniae* nasopharyngeal colonization (>10 000 genome copies/mL), *M. pneumoniae* positivity, or NPS virus positivity

We feel that these outcomes are important to children and their caregivers, especially in mild illness with an excellent prognosis. These will mostly be participant-report or caregiver-report measures, and will be measured through participant documentation in the diaries; there will also be secondary verification by the research assistant (RA) at telephone contact on days 3–5 and 7–10 after enrolment. Microbiology outcomes will be measured through intestinal sampling at baseline, 14–21 days post-enrolment, and 3–6 months post-enrolment (see below).

### Study procedures

#### Screening, enrolment, and randomization

Attempted recruitment will be triggered in the ED as soon as a child of the appropriate age is diagnosed with CAP by the ED physician during study hours. All participants will be provided with the appropriate medications and nasopharyngeal, salivary, and enteric specimens will be collected; note that participants can opt out of providing any of these specimens and still participate in the trial. The interpretation of the chest radiograph by the radiologist will be abstracted and recorded.

All eligible participants will be assigned a study ID. Randomization of the participants will be completed using a randomization scheme (stratified by site) generated by the study pharmacy at MCH using a random number generator, using block sizes of 2 and 4, and will pre-assign kit contents to a given study ID. The RA will notify the site study pharmacy after a successful recruitment and will communicate the study ID so that the appropriate medications can be prepared by the research pharmacist; for the duration of the study, the pharmacist will be the only individual not blinded to the participant’s treatment allocation. All participants will be given an initial 5 days of amoxicillin to begin. For potential participants at MCH who present at a time when an RA is not available, the attending ED physician will prescribe amoxicillin at the study dose (i.e. the standard of care) and obtain consent for subsequent RA contact and potential enrolment within 24 h after ED discharge. Recruitment at CHEO will only proceed between 10.00 a.m. and 10.00 p.m. daily. Participants will be given all study medications at enrolment unless a participant presents outside of pharmacy operational hours at MCH; in that instance, the RA will dispense an after-hours open-label amoxicillin kit for the first 5 days of treatment to the caregiver and contact the pharmacy with the study ID so that the blinded medication kit can be prepared and provided to the caregiver at a later date (prior to day 6).

#### Follow up

The RA will phone the caregiver once at day 3–5 and once at day 7–10 to verify the clinical stability of the participant. Caregivers will also be explicitly informed that they can contact the study team in the event of a concern prior to the scheduled phone calls. The caregiver will be given a study diary to complete each day, which will include the following: temperature, dyspnoea (older participants), increased work of breathing, school attendance, caregiver absenteeism from work duties, days of mild diarrhoea, mild (abdominal discomfort, rash) and severe (anaphylaxis) drug adverse reactions, and the number of missed medication doses, if applicable. The caregivers will be instructed how to take their child’s temperature and assess increased work of breathing. Any participant who has persistent fever more than 3 days post-enrolment will receive an additional 5 days of open-label amoxicillin after the initial 5 days (i.e. will receive a guaranteed 10 days of antimicrobial agents). Any participant who worsens clinically subsequent to enrolment at any time point prior to the primary outcome visit will be urged to seek medical care without delay either at the site where recruitment took place or with another physician (e.g. family physician, walk-in clinic); all participants will be provided with printed documentation about the SAFER trial and contact information for the local Principal Investigator (PI) for the benefit of the medical professional re-evaluating the participant. The criteria for permanent discontinuation of the experimental aspect of the study protocol in an individual subject are as follows: failure to defervesce by day 4 post-enrolment; requirement for hospitalization because of worsening respiratory illness; severe reaction potentially associated with amoxicillin, such as a generalized allergic reaction (urticarial rash, bronchospasm, angioedema, hypotension, etc.); requirement for prohibited concomitant medications; completion of treatment/intervention as defined by the protocol; or clinical reasons believed by the physician to be life-threatening.

The daily symptom diary will include prompts to aid caregivers to remember the three daily doses of study medication. The RAs will also encourage optimal adherence to medication administration at baseline and at each of the two scheduled follow-up phone communications.

**Fig. 1:**
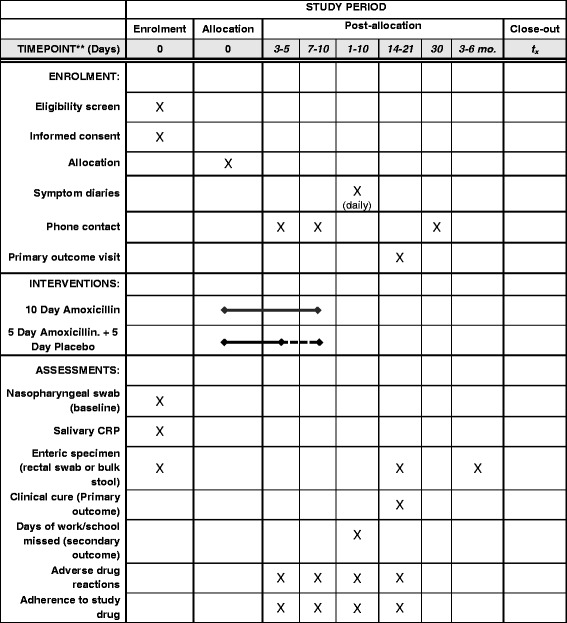
SPIRIT diagram showing participant involvement. *CRP* C-reactive protein

The participant will return to the hospital at day 14–21 for the measurement of the primary outcome and to deliver the study diaries. At this visit, the RA will verify whether the participant developed new fever or required additional antimicrobials, and will have a physician or nurse perform a brief physical examination (temperature, respiratory rate, and assessment of increased work of breathing). The medication bottles will be returned and the volume of remaining medication determined. The RA will phone the caregiver one month after enrolment to verify continued clinical stability. All attempts will be made to follow up participants, even those who are already known to represent clinical failure (e.g. do not defervesce by day 4 post-recruitment and receive 10 days of open-label amoxicillin) or who receive additional interventions outside of the study (e.g. have additional antimicrobial agents prescribed by other clinicians) to ensure completeness of data collection (Fig. 1).

#### Unblinding

There are three scenarios that may lead to unblinding of treatment assignment:

1. Clinical deterioration in the patient on day 6 or later after randomization, possibly consistent with worsening CAP, or
2. The occurrence of a clinically significant adverse event plausibly related to amoxicillin administration on day 6 or later after randomization
3. Caregiver-initiated withdrawal of the participant from the study at day 6 or later up until the clinical evaluation at day 14–21, if requested by the caregiver

When the RA is made aware of participant difficulties consistent with scenario 1 or 2, the RA will inform the local PI or delegate, who may recommend participant re-evaluation, either in the ED or in a hospital clinic where another study physician can evaluate the participant properly. If unblinding is required, this information can be provided to the physician evaluating the study participant following authorization by the local PI or their delegate.

### Laboratory testing

#### Microbiology testing

All nasopharyngeal specimens acquired at baseline will be tested for the following:

1. Eleven different respiratory viral pathogens (influenza A, influenza B, RSV A/B, parainfluenza virus I-III, rhinovirus, enterovirus, adenovirus, and human metapneumovirus) using the laboratory-developed multiplex PCR platform in clinical use at McMaster
2. Three different atypical respiratory pathogens (*Legionella pneumophila, M. pneumoniae,* and *Chlamydophila pneumoniae*) using a multiplex loop-mediated isothermal amplification (LAMP) platform
3. *S. pneumoniae* incidence density (uniplex laboratory-developed PCR using a *lytA* target)

Enteric samples will be requested to establish baseline intestinal microbiome composition and to assess colonization with antibiotic resistant organisms (AROs), namely ampicillin-resistant *Escherichia coli* and *Enterobacteriaceae* with extended-spectrum beta-lactamase (ESBL) production. These samples will either be bulk stool or rectal swabs collected using flocked rectal swabs (Copan Italia S.A.) and eluted using Cary Blair and/or eNAT transport medium. Enteric samples can be collected up to 24 h post-visit, as necessary. Another enteric sample will be collected during or immediately subsequent to the primary outcome visit. Finally, caregivers will be asked to collect a final set of enteric samples for intestinal microbiome analysis and/or ARO colonization measurement 3–6 months post-enrolment. All laboratory specimens will be stored at -80 °C until testing occurs. Enteric specimens will have 16S ribosomal RNA (rRNA) profiling conducted, with baseline samples compared to samples collected at the primary outcome visit and then later at 3–6 months post-enrolment.

#### Other testing

If blood work was ordered by the attending ED physician, results of complete blood count and/or CRP will be noted but no additional blood draws will be taken as part of study activities. For those participants who did not have serum CRP measured, an optional salivary sample will be requested for salivary CRP measurement; this will be acquired using a small sponge (Salimetrics Children’s Sponge) placed in the buccal mucosa or beneath the tongue (for 60–90 seconds).

### Data handling

Data will be stored electronically in the secure Research Electronic Data Capture (REDCap) web-based platform on a server hosted at the Department of Pediatrics at McMaster University. Only research team members will have access to the data platform, and each team member will be granted access through a secure login by the study PI or his proxy. All entries will be double-checked, ranges will be checked for selected covariates, and quality will be assessed periodically using custom data queries. All subject-related information including case report forms, laboratory specimens, evaluation forms, reports, etc. will be kept strictly confidential. All records will be kept in a secure, locked location and only research staff will have access to the records. Subjects will be identified only by means of a coded number specific to each subject. As noted above, the REDCap study database is securely protected and encrypted. All computerized databases will identify subjects by numeric codes only. Upon request, subject records will be made available to the study sponsor, monitoring groups representative of the study sponsor, and Health Canada.

### Safety and adverse events

The investigators will report adverse events (AEs) as per standard procedure of the Hamilton Integrated Research Ethics Board (REB), the CHEO REB, and Health Canada. Specific adverse events that have a higher likelihood of being caused by amoxicillin administration include rash, diarrhoea, candidiasis, and anaphylaxis. Nausea and vomiting are commonly reported in association with amoxicillin use, though a recent meta-analysis did not find their occurrence to be any more common with amoxicillin use as compared to placebo [36]. As rash, diarrhoea, nausea, and vomiting can also be caused by inter-current infection, each participant reporting an AE will have to have their case reviewed by an investigator. Parents will be informed that any development of anaphylaxis requires immediate evaluation at the appropriate ED; should this happen while the participant is taking the second set of medication or shortly thereafter (i.e. day 6–14) the participant’s treatment assignment will be unblinded if deemed necessary per the attending physician and local PI/delegate. Anaphylaxis that occurs more than 24 h after cessation of amoxicillin (or placebo) is highly unlikely to be related to that product.

We note that adverse events associated with antimicrobials are likely to be fewer in study participants than in children whose caregivers do not agree to participate in the study; furthermore, the AEs that occur are likely to be handled more promptly. This is because the study drug we are using is the standard of care for paediatric CAP and the entire aim of the study is to verify that shorter courses of amoxicillin are non-inferior to amoxicillin given for the standard longer duration. As noted previously, we will be actively seeking drug AEs during the phone calls at day 3–5 and 7–10 and will be asking parents to fill out a daily symptom diary that will ask about potential AEs.

Specific adverse events that have a higher likelihood of being caused by short durations of antimicrobial therapy for CAP include recrudescence of CAP and symptoms associated with this, including fever, cough, difficulty breathing, tachypnoea, abdominal pain, and malaise. Having stated this, it is also not uncommon for children to experience these symptoms when contracting a new (inter-current) respiratory viral infection. We will be actively seeking evidence of potentially recrudescent infection when contacting caregivers at day 3–5 and day 7–10; additionally, we will ask caregivers to contact us if these symptoms develop. Any participant with new or worsening respiratory symptoms will have their case reviewed by the local PI and urged to see a physician, if necessary; should this physician feel that the participant might have inadequately treated bacterial CAP, and the PI concurs that unblinding will be beneficial to patient management, the participant’s treatment assignment will be unblinded.

We do not expect any AEs associated with study-related specimen collection. Nasopharyngeal swabs are routinely done at both study sites in all children admitted to hospital with a respiratory syndrome. Rectal swabs are similarly very low-risk interventions; the procedure for obtaining a rectal specimen is similar to that involved in taking rectal temperature (the reference standard for temperature measurement in young children); however, obtaining a rectal swab specimen takes much less time than rectal temperature measurement. Any AE that occurs between the times a study participant signs the informed consent form and the time the participant departs the study at the end of the final follow-up visit (or at the time of early discontinuation of the subject from the study for any reason) will be captured and recorded. AEs will be described as “pre-intervention”, “‘related”, and “serious” as applicable, but all will be recorded. Physician diagnoses of AEs as a particular syndrome (e.g. “cellulitis”) will be recorded.

All serious AEs (SAEs) that are both unexpected and related to the study product will be reported to Health Canada, and those related to the study product or procedures will be reported to the appropriate REB as soon as possible if required by the REB, but no later than 15 calendar days (7 days if fatal or life-threatening) after first knowledge of the event. Further information and significant new information on ongoing reported SAEs will be provided to the sponsor, the REB and Health Canada, as applicable. Copies of all information about the SAE will be kept in the regulatory binder.

All caregivers will be asked to complete a symptom diary form each day post-randomization until the tenth post-randomization day; questions on this form will seek information on clinical features of persistent or worsening respiratory disease (e.g. fever, difficulty breathing, overall clinical status) in addition to common (e.g. diarrhoea) and serious (e.g. anaphylaxis) AEs known to be associated with amoxicillin administration. In addition, the RA will specifically ask about symptoms and signs consistent with respiratory deterioration or amoxicillin-associated AEs. This will also be done at the time of visit 1, when the participants return to be clinically evaluated at day 14–21 post-randomization.

The SAE most likely to occur is hospitalization in the first few days post-recruitment, due to worsening respiratory status. Though the majority of children with mild CAP respond well to oral antimicrobial therapy, a small percentage will worsen and require admission to hospital because of progressive oxygenation failure and/or the need for operative drainage of pleural-based collections. These SAEs would not be related to any trial procedures, as all study participants will be receiving the first-line antimicrobial agent of choice (high-dose amoxicillin) for the first 5 days post-enrolment. We note that the risk of harm to trial participants will likely be less than to non-participants, because study staff will be contacting all study participants at intervals to verify clinical stability. As detailed above, the RAs will contact all study participants once at day 3–5 post-recruitment and once at day 7–10 post-recruitment and will be actively questioned about symptoms indicative of worsening respiratory status. Furthermore, all participants that are persistently febrile at day 4 post-recruitment will complete 10 days of open-label high-dose amoxicillin.

### Statistical analysis

#### Sample size

We estimate the baseline failure rate of standard therapy to be ~ 5%; this estimate is consistent with previous studies in children [13], is less than in similar adult studies [7], and is the approximate rate that was seen in the pilot study. We will use a non-inferiority margin of an additional 7.5%. As this is a non-inferiority trial, the crucial statistical comparison will be between the 97.5% (one-sided) CI of the difference between the failure rate of the experimental arm and the standard therapy arm; should the upper bound of this difference be smaller than 7.5%, a conclusion of non-inferiority will be reached. As the maximum baseline failure rate in the reference arm is probably 5%, the maximum failure rate in the experimental arm that would still be felt to be “non-inferior” would be 12.5%; the margin of 7.5% was selected to make the maximum failure rate in the experimental arm less than 13.5%, the median acceptable failure rate in the treatment of CAP identified in a survey of infectious-disease physicians [37]. Setting α at 5%, with 80% power, 135 participants in each arm will be required for this trial (PASS software package, NCSS LLC, Kayesville, UT, USA); as we will have accrued ~ 60 subjects in the pilot to be “rolled over”, an additional 210 participants will be required. Our experience conducting the pilot, combined with an analysis of visits to the EDs of the study sites, leads us to believe that this target should be achievable. It is estimated that the target sample size should be attainable after 2 years of enrolment.

#### Analysis

We will use descriptive statistics to describe the baseline characteristics of the groups reported as count (percent) for categorical variables, and mean (standard deviation) or median (first quartile, third quartile) for continuous variables, depending on the distribution. We will adopt Consolidated Standards of Reporting Trials (CONSORT) criteria for reporting on non-inferiority and equivalence trials in reporting the trial [38]. The principal analysis will be per- protocol, as is recommended for non-inferiority trials [39–41]. The principal analysis is not intention-to-treat (ITT) simply because the effect of ITT analysis is to reduce the difference seen between treatment groups; in a superiority trial, ITT analysis functions to buttress a conclusion of superiority, but in a non-inferiority trial it could lead to a false conclusion of non-inferiority by masking a true difference between treatment arms.

We will use logistic regression to analyze binary outcomes, Poisson regression for count outcomes and linear regression for continuous outcomes. Descriptive analyses will be used to compare rates of viral and atypical co-infections in the entire study population and between groups.

We will perform some subgroup analyses by adding an interaction term between treatment group (5-day vs. 10-day course) and the following subgroups: (1) older (age 5–10 years) vs. younger (age <5 years); (2) higher vs. lower salivary CRP; and (3) virus/*Mycoplasma* detected in baseline NPS vs. no virus detected. These interaction tests will be exploratory in nature and not adjusted for multiple testing [42].

These analyses will be exploratory. The following sensitivity analyses are planned: (1) intention-to-treat analysis; (2) strict per-protocol analysis including only those participants who were adherent to their medications and whose radiographs were reported by a radiologist to demonstrate alveolar infiltrates; (3) per-protocol analysis stratified by whether the saliva CRP was greater than the 75th percentile; and (4) per-protocol analysis stratified by whether a virus, an atypical pathogen, or high-level *S. pneumoniae* colonization was found in the NPS. If evidence is found of effect modification or confounding related to the above parameters additional analyses will be undertaken. The results of all analyses will be reported as estimate of effect, corresponding 95% CI and associated *p* values. All *p* values will be reported to three decimal places with those less than 0.001 reported as *p* < 0.001. The criterion for statistical significance will be set at alpha = 0.05. Table 1 provides a summary of the outcomes, corresponding hypotheses, measure, and method of analysis.

**Table 1:** Variables, measures, and methods of analysis

Variable/outcome	Hypothesis	Outcome measure	Methods of analysis
Primary			
Clinical cure (per-protocol)	Short-course (5-day) treatment with high-dose amoxicillin is non-inferior to long-course (10-day) treatment	At visit (day 14–21) composite of: 1. Defervesced on or before day 4 2. No more than 1 further fever spike until visit 3. No tachypnoea and decreased work of breathing 4. No additional antibacterials given Dichotomous	Logistic regression
Secondary, between-group comparisons			
Absenteeism (caregiver, from work)	Short-course treatment is non-inferior to long-course treatment	Daily symptom diaries: self-reported number of caregiver-days absent from work (count)	Poisson regression
Absenteeism (child, from daycare/school)	Short-course treatment is non-inferior to long-course treatment	Daily symptom diaries: reported number of child-days absent from daycare/school (count)	Poisson regression
Mild drug adverse reactions	Fewer in short-course arm than in long-course arm	Daily symptom diaries: Reported number of child-days with diarrhoea, rash, abdominal pain, yeast infection (count)	Poisson regression
Anaphylaxis and other severe drug adverse reactions	Fewer in short-course arm than in long-course arm^a^	Daily symptom diaries, SAE reports. Dichotomous	Descriptive statistics
Adherence to study medications	Better in short-course arm than in long-course arm	Daily symptom diaries, RA interview: “adherence” defined as > 80% amoxicillin doses given (<3 doses of initial 15 doses in short-course arm, < 6 doses in reference arm) Dichotomous	Logistic regression
Recurrence of respiratory illness after primary outcome visit but before 30-day follow up	Short-course treatment is non-inferior to long-course treatment	RA interview. Dichotomous	Logistic regression
Development of antibiotic-resistant organism (ARO) colonization	Less frequent in short-course arm than in long-course arm	Enteric swab testing: “new colonization”’ defined as 3–6 month swab ARO positivity in context of baseline swab ARO negativity. Dichotomous	Logistic regression
Disruption of gut microbiome	Less marked in short-course arm than in long-course arm	Enteric swab testing: comparison of gut microbiome at 3–6 months to baseline gut microbiome. Continuous	Linear regression
Tertiary, entire-cohort			
Distribution of salivary C-reactive protein in cohort	Mean will be greater than that observed in children with bronchiolitis but less than that observed in children with empyema	Salivary swab testing: continuous	Descriptive statistics expressed as mean (95% CI)
Prevalence of high-level *S. pneumoniae* nasopharyngeal colonization	Majority of cohort will be positive	Nasopharnygeal swab (NPS) testing: Dichotomous	Percentage, with 95% CI
Prevalence of *Mycoplasma* detection	Minority of cohort will be positive	NPS testing: Dichotomous	Percentage, with 95% CI
Subgroup analyses			
Older (age 5–10 years) vs. younger (age <5 years)	Older age group will have higher salivary CRP values, lower rates of *S. pneumoniae* high-level colonization, more *Mycoplasma* positivity, and decreased rates of clinical cure	Clinical cure (as defined above)	Logistic regression with an interaction term between subgroup and treatment variables
Higher vs. lower salivary CRP	Higher salivary CRP will be associated with decreased rates of clinical cure	Clinical cure	Logistic regression with an interaction term between subgroup and treatment variables
Virus/*Mycoplasma* detected in baseline NPS vs. no virus detected (for primary outcome)	Detection of a virus or *Mycoplasma* will not affect observed rates of clinical cure	Clinical cure	Logistic regression with an interaction term between subgroup and treatment variables
Sensitivity analyses – for primary outcome only			
Intention-to-treat	Results will remain robust	Clinical cure	Logistic regression
Strict per-protocol (those with adherence >80% and radiologist-verified pneumonia)	Results will remain robust	Clinical cure	Logistic regression

*SAE* serious adverse event, *RA* Research Assistant, *ARO* antibiotic-resistant organism, *CRP* C-reactive protein

^a^Too few events expected to be able to observe differences in treatment arms

#### Interim analysis

The Data Safety Monitoring Board (DSMB), comprising a biostatistician and two clinician-investigators completely independent of the sponsor and funders, will oversee a single interim analysis of the study data halfway through enrolment (i.e. after 100 participants have been enrolled). Rates of early clinical failure (the primary outcome) will be calculated for each arm of the study and the study will be prematurely terminated if the proportion of treatment failures in the experimental arm is statistically significantly greater (*p* < 0.0001) than 7.5% *more* than the proportion of treatment failures in the control arm. Should one of the arms of the trial be found to be this much greater than the other, the DSMB will order unblinding, and should the increased failure rate be in the control arm, the trial will continue, as this would certainly be due to chance. The trial will not be stopped early for benefit simply because trials stopped early for benefit have been shown to consistently overestimate treatment effects [18], and, if short-course therapy is truly non-inferior to standard therapy, participants in the trial would be at overall decreased risk compared with non-participants due to the surveillance measures built into the trial. Additionally, the DSMB will review safety data on a biannual basis for each arm of the study; specific items that will be monitored include the number of participants in each arm that clinically worsen on or after 6-days post recruitment and require a change in antimicrobial therapy; the number of participants in each arm that develop an SAE; and the number of participants in each arm that clinically worsen after the primary outcome measurement and require institution of antimicrobial therapy. If safety concerns arise, more frequent meetings will be initiated, and the trial may be terminated. The DSMB will receive immediate notification and reports of serious adverse reactions related to study procedures or medications.

### Study monitoring

On-site monitoring of the MCH site and the CHEO site, both tertiary-care children’s hospitals, will be conducted by qualified research personnel (the main study PI, the main study research coordinator, or quality assurance personnel) as required. Monitoring will be conducted through personal visits with the local PI and site staff (every 3–6 months or as needed based on enrolment and participant study visits) as well as any appropriate communications by mail, fax, e-mail, or telephone. The purpose of monitoring is to ensure compliance with the protocol and the quality and integrity of the data. The essential documents in the investigator regulatory files will be monitored and checked for accuracy and completeness. The monitor will identify any items missing from the regulatory binder. Site personnel are responsible for maintenance of the regulatory binder. The consent document will be reviewed for content to ensure it contains the required (and additional, as applicable) regulatory elements. The consent document will be compared to the protocol and site-specific REB procedures for informed consent documentation to ensure agreement between the two documents. Consent form monitoring will be documented in the monitoring time point report.

#### Knowledge translation (KT) plan

The nature of the proposed trial is strongly toward the pragmatic end of the clinical trial spectrum [43]; consequently, the results of the trial will be positioned for rapid integration into clinical practice by Canadian physicians. Research team members will collaborate with established networks of clinicians experienced in the dissemination of clinical guidelines to healthcare practitioners, i.e. the Canadian Paediatric Society, Association of Medical Microbiology and Infectious Disease Canada, Infectious Disease Society of America, Canadian Association of Emergency Physicians, and Pediatric Emergency Research Canada (PERC). Given that PERC, a highly successful research network involving 15 children’s hospitals, represents a key group of knowledge users for this study, the executive was invited to the table in the design phase to ensure that the study objectives were relevant to Canadian emergency physicians and the study protocol was structured in such a way to optimize both internal and external validity. PERC has since unanimously endorsed the proposed study as one deserving of its support. The above-noted collaborations will be stimulated though presentation at major Canadian and American meetings (Pediatric Academic Societies, Canadian Paediatric Society, etc.); healthcare decision makers will be provided a one-page synopsis of the results and invited to meet with study team members to discuss the implications. The end-of-grant KT strategy will also focus on publication of results in a peer-reviewed open-source journal (preferably a general paediatric journal because of the broad audience), oral and poster presentation at local and national meetings, and leveraging dissemination through the diverse professional networks of the research team members (the applicant and co-investigators are trained in disciplines including paediatric infectious disease, adult infectious disease, medical microbiology, clinical epidemiology, and paediatric emergency medicine). In all cases, messages will be tailored to ensure relevance to the target audience.

## Discussion

SAFER is a pragmatic randomized controlled trial designed to embrace the conditions of real-world emergency medicine practice and “bridge the gap”’ between research and care. The pragmatic nature of our study will ideally position the results for rapid integration into clinical practice by Canadian physicians.

As is the case for any randomized controlled trial (RCT), there are aspects to its design which are not optimal. One issue is that should children without bacterial pneumonia be included, statistical “noise” would be introduced, which might mask a true “signal” indicating that 5 days was in fact inferior to 10 days of high-dose amoxicillin (type I error). Unfortunately, there are no consensus criteria (clinical, radiologic, or otherwise) for the diagnosis of pneumonia, and so all CAP trials must construct a definition that appears “appropriate”. Our definition is almost identical to the “reference standard” in a recent study designed to investigate the accuracy of ICD-9-CM billing codes [44] and very similar to those used in other clinical trials [45–47] (many studies of pneumonia simply use clinician diagnosis as a definition [48–50], which is often imprecise and subjective). The inclusion of fever as a necessary criterion will diminish the probability of recruiting participants with pertussis, which is much less likely to be associated with fever [51], or non-infectious conditions. The necessity for participants to display a respiratory symptom or sign will diminish the probability of recruiting those with infections of other organ systems who are erroneously diagnosed with pneumonia. The requirement for participants to have a chest radiograph displaying a pneumonic infiltrate will likely increase the probability that they have an infection caused by a bacterial pathogen. Finally, since the aim of this pragmatic trial is to answer a real-world question asked by emergency physicians, it is important that all study participants are actually diagnosed with CAP. Unfortunately, none of the above is sufficient to reliably distinguish viral from bacterial pneumonia. It is very difficult to reliably determine the microbiologic aetiology of paediatric CAP without percutaneous lung biopsy, which has been applied in some settings [52], but is not practical in North America. Gram staining/culturing of sputum can help identify the aetiology of adult CAP but the difficulty of obtaining an adequate sputum sample from a young child renders it effectively useless in the paediatric ED. Culture or PCR of nasopharyngeal swabs (NPS) can readily detect *S. pneumoniae*, but its detection has not yet been shown to be specific for the diagnosis of pneumonia, as colonization was previously found to be common in young children [25]. Furthermore, preschoolers commonly develop viral pneumonia [5]. A recent, large multicentre prospective cohort study demonstrated that 73% of children admitted to hospital with severe pneumonia in the USA in 2010–12 had a detectable respiratory viral pathogen, compared to only 15% in whom a bacterial pathogen could be found [53]. Many centres routinely use multiplexed PCR panels that can detect almost all common important respiratory viruses in NPSs with high sensitivity and specificity [54]. However, it should be emphasized that the detection of a respiratory virus in an NPS does not rule out bacterial co-infection, a phenomenon that appears to be relatively common [55]. Given the extreme difficulty in discerning between viral infection and viral and bacterial co-infection, it should not be surprising that radiographic criteria for distinguishing between viral and bacterial pneumonia have never been developed, though many clinicians would presume that a child who had an alveolar infiltrate on chest radiograph would have a bacterial pulmonary infection. Other investigators have used CRP or white blood cell (WBC) count cutoffs to minimize the probability of recruiting patients without bacterial pneumonia; however, not only is there substantial overlap between the CRP/WBC distributions in children with viral and bacterial pneumonia, but in many regions blood work is rarely requested for children without severe pneumonia. Consequently, insistence on this testing would bring about unnecessary inconvenience and pain for participants and their families; furthermore, an algorithm that required blood work to stratify the severity of CAP would limit the usefulness of this testing in a real-world scenario. The SAFER trial is attempting to provide high-quality evidence for the management of children encountered on an everyday basis by clinicians working in North American EDs, and so inclusion of some children who may not have bacterial disease will not limit the generalizability of the results of the study.

The selection of the non-inferiority margin has a significant impact on trial outcome. It is clear that clinical considerations should inform the determination of the margin; if the margin is judged as unacceptably wide to clinicians, a “positive” non-inferiority trial will have little impact on practice. However, selection of the margin based solely on the clinical experience of the principal investigator should probably be avoided, despite how common this has been in the past. The US Food and Drug Administration (FDA) has provided guidance relating to statistical considerations affecting the selection of the non-inferiority margin [39]; note that their main concern seems to be that if the experimental treatment is found to be non-inferior to the standard therapy, there is sufficient evidence to be reasonably confident the experimental treatment is also superior to placebo [39]. The FDA draft guideline first instructs investigators to review meta-analyses of clinical trials of the standard therapy compared to placebo to discern the pooled estimate of efficacy; if only single trials are available, they can be used, but there will be much lower precision in the estimates of efficacy. Unfortunately, we cannot follow this guidance for the statistical determination of an appropriate margin for a trial such as SAFER because we do not have reliable data about the failure rate of placebo treatment of bacterial pneumonia in children living in the modern era. Estimates of child CAP-specific mortality from the pre-antibiotic era cannot be used since this would without doubt overestimate CAP-specific mortality in children today.

Adherence to medication is, in general, difficult to ensure. Caregivers of participants in this trial will be reminded far more frequently of the need to administer medication (via RA reminders and study diaries) than they would otherwise be; our adherence measures are similar to those in other major recent RCTs comparing short-course and standard-course antimicrobial therapy in children [56]. Despite this, it is entirely possible that adherence will be suboptimal, which could also predispose to a false conclusion of non-inferiority, especially if adherence to medication drops off after 5 days. From a practical standpoint, this is still important information for clinicians managing real patients, and would not greatly affect the generalizability of the results of the study (Additional file 1).

### Trial status

Actively recruiting since August 2016 using protocol v5.0 (date 12 August 2016).

## Additional file

Additional file 1: SPIRIT Checklist: Recommended items to address in a clinical trial protocol. (DOC 122 kb)

## Abbreviations

ARO: Antibiotic-resistant organism
CAP: Community-acquired pneumonia
CHEO: Children’s Hospital of Eastern Ontario
CRP: C-reactive protein
DSMB: Data Safety Monitoring Board
ED: Emergency Department
FDA: Food and Drug Administration
HIV: Human immunodeficiency virus
IDSA: Infectious Disease Society of America
ITT: Intention-to-treat
MCH: McMaster Children’s Hospital
NPS: Nasopharyngeal swab
PERC: Pediatric Emergency Research Canada
PI: Principal Investigator
RA: Research Assistant
RCT: Randomized controlled trial
REB: Research Ethics Board
WBC: White blood cell
